# The impact of bullying cognition on school bullying among Chinese primary school students: a moderated mediation model of resilience and sex

**DOI:** 10.3389/fpubh.2024.1470322

**Published:** 2024-10-16

**Authors:** Liping Fei, Tianwen Li, Yongli Li, Maoxu Liao, Xin Li, Yiting Chen, Rong Zhang

**Affiliations:** ^1^Department of Epidemiology and Health Statistics, School of Public Health, Southwest Medical University, Luzhou, China; ^2^Information and Education Technology Center, Southwest Medical University, Luzhou, China

**Keywords:** school bullying, cognition, psychological resilience, primary school students, mediating effect, moderating effect

## Abstract

**Background:**

Previous studies have shown the positive effects of bullying cognition on school bullying behavior among young people, but the mechanism underlying this association remains unclear.

**Methods:**

We conducted a cross-sectional study with 5,903 primary school students in grades 3–5 in Luzhou city. Hayes’ PROCESS macro was used to test the mediating effect of psychological resilience and the moderating effect of sex on the relationship between bullying cognition and school bullying after controlling for grade.

**Results:**

Psychological resilience partially mediated the relationship between school bullying cognition and victimization (*β* = −0.0174, 95% CI: −0.0219 to −0.0132) and between perpetration (*β* = −0.0079, 95% CI: −0.0104 to −0.0055). This study revealed that sex moderated the relationship between school bullying cognition and perpetration (*β* = 0.0383, *p* < 0.001) and victimization behavior (*β* = 0.0400, *p* < 0.001).

**Conclusion:**

These findings suggest that it is crucial for education regulators, schools, and families to cultivate students’ school bullying cognitions and psychological resilience, which may help to decrease the prevalence of school bullying. Especially for boys, improving their bullying cognition may largely decrease its perpetration.

## 1 Introduction

Bullying is an intentional aggressive action that repeatedly takes place among two or more people with an asymmetric power relationship (1). Bullying behavior can be divided into three types: direct physical bullying, direct verbal bullying and indirect bullying (2). Participants involved in school bullying include perpetrators (only bullying others), victims (only being bullied), and perpetrator-victims (both bullying and being bullied) (3). A meta-analysis of 68 studies showed a pooled overall prevalence of bullying victimization of 22.7% and a pooled overall prevalence of bullying perpetration of 15.7% among the Chinese school-age population (4). Recent surveys conducted in Wuhan and Xinjiang in China report prevalence of bullying among primary school students at 24.2% (5) and 26.1% (6), respectively. Studies have shown that school bullying can affect pupils’ academic careers, mental health, and further behaviors (7, 8). Given the high prevalence of bullying among primary school students and its profound implications for their future, it is essential to develop a comprehensive understanding of its mechanisms to inform the creation of targeted intervention measures.

Cognition is the process of acquiring knowledge, applying knowledge, or processing information (9). According to cognitive behavior theory (10), there is a close relationship between cognition and individual behavior. Students’ cognitions of school bullying also impact the occurrence of school bullying. For example, students who believe that “forces are a symbol of power and that fists are more effective than words” are more likely to be the perpetrators of school bullying, and students who share this view are also more likely to be victims of school bullying (11). School bullying is also more prevalent when students perceive it as a normal behavior (12). Individuals with a low level of cognition about school bullying are more likely to ignore the occurrence of bullying or even accept the occurrence of school bullying through observation and learning and eventually change from victims of school bullying to perpetrators (13). Correcting inappropriate cognition can effectively prevent aggressive and bullying behaviors among children and adolescents (14, 15). Misunderstanding or lack of knowledge of bullying was established to be closely related to the occurrence of school bullying in China (16). Thus, we propose Hypothesis 1: Improving cognition of school bullying reduces its occurrence.

Resilience is also an important influencing factor of school bullying (17–19). Resilience is the ability to help individuals adapt well and recover from severe stress, frustration and adversity (20). Previous studies have shown that there is a close relationship between psychological resilience and school bullying and that psychological resilience can predict the occurrence of school bullying (21). According to the mental defense mechanism (22), psychological resilience can reduce the adverse effects of school bullying (23). Students with a higher level of resilience can show better coping and adaptability when dealing with difficulties and exhibit less bullying behavior. Psychological resilience was negatively associated with school bullying. In addition, previous studies have shown that there is a significant positive correlation between the level of resilience and school bullying cognition (24, 25). A study showed that there is a close relationship between bullying cognition and psychological resilience and between psychological resilience and school bullying (18); however, it is unknown whether psychological resilience mediates the relationship between bullying cognition and school bullying. Like in related studies, psychological resilience can adjust the relationship between school bullying victims and self-harm, reduce the self-harm behavior of those who suffer from school bullying (26), and adjust the relationship between school bullying and mental health (27). Factors such as a good family atmosphere and social support can also reduce symptoms of bullying by improving the level of psychological resilience (28, 29). The greater the level of school bullying cognition is, the greater the level of psychological resilience (25). Studies have also shown that psychological resilience is negatively associated with school bullying behaviors, including perpetration and victimization. Students who experience bullying behaviors often experience anxiety, fear and depression, and increased psychological resilience is better able to mitigate negative psychological effects and adopt more positive behaviors and coping styles to reduce school bullying (30). Therefore, we propose Hypothesis 2: Improving cognition of school bullying positively affects psychological resilience and thus reduces the occurrence of school bullying. Resilience mediates the relationship between cognition and school bullying.

There are sex differences in cognition, behavior, and resilience. Many studies have shown that there are differences in the prevalence of school bullying between different sex groups and that boys are more likely to become involved in bullying than girls are (31, 32). The level of cognition of school bullying is also greater for girls than for boys (33). There are also sex differences in resilience (34, 35). However, the relationships among students’ cognitions of school bullying, school bullying behavior and sex are unclear. This study proposes Hypothesis 3 that sex plays a regulatory role in the prediction of school bullying.

**Figure 1:**
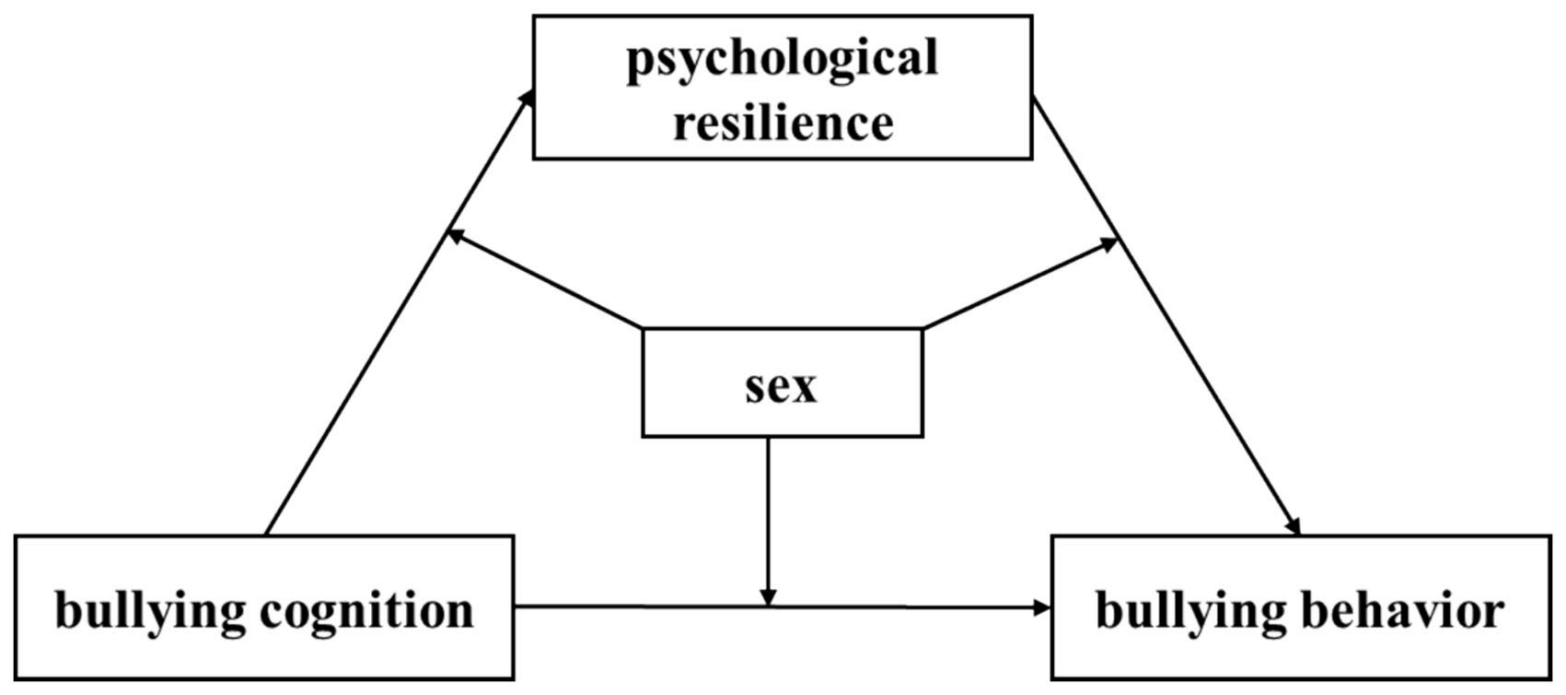
A constructed moderated mediation model.

The elementary school period is a crucial time for developing behavioral habits and mental wellness and serves as a sensitive phase for addressing negative behavioral patterns/remedying undesirable behaviors. Additionally, studies on school bullying in China have focused primarily on secondary school students (36–38), with limited research conducted on elementary school students. Furthermore, even fewer studies have investigated the associations among psychological resilience, perceptions of bullying, and bullying behavior among elementary school students. Therefore, a moderated mediation model (Figure 1) was constructed in this study to explain the influence of bullying cognition on bullying behavior and the underlying mechanism of psychological resilience and sex. This study aimed to provide a theoretical perspective on the factors underlying bullying behavior and to propose methods for preventing and reducing school bullying among elementary school students.

## 2 Methods

### 2.1 Participants

From October to November 2019, primary school students in Luzhou were selected by stratified cluster sampling as a representative sample for the questionnaire survey. Based on the gross domestic product (GDP) ranking of all districts in Luzhou in 2018, the seven districts of Luzhou City were categorized into three groups: high GDP districts (Jiangyang and Luxian), medium GDP districts (Longmatan and Hejiang), and low GDP districts (Naxi, Gulin, and Xuyong). First, one district was randomly selected from each group: specifically, Jiangyang from the high GDP group, Longmatan from the medium GDP group, and Naxi from the low GDP group. Next, within each selected district, one private elementary school and three public elementary schools were randomly chosen. Finally, four classes from the 3rd, 4th, and 5th grades of each selected school were randomly drawn, with all students from these classes included as the participants. Students were informed that the questionnaires were anonymous and confidential and that there were no correct or wrong answers to any of the questions. All the questionnaires were administered by trained interviewers in the absence of a teacher. A total of 6,066 subjects were obtained. After excluding subjects who returned questionnaires with 5 or more missing values, a total of 5,903 subjects were included in the analysis. This study was approved by the Medical Ethics Committee at the Affiliated Hospital of Southwest Medical University (NO. KY2019128), and all the students’ parents signed informed consent forms.

### 2.2 Instrumentation

Using preexisting and validated measures, a questionnaire was constructed to collect the data. The survey included four dimensions—demographic information (sex, grade), level of cognition about school bullying, level of psychological resilience and occurrence of school bullying.

#### 2.2.1 School bullying cognition level

In this study, school bullying cognition is operationally defined as the thoughts, beliefs, and attitudes pertaining to bullying behaviors, specifically one’s stance toward supporting or opposing specific instances of bullying. The School Bullying Cognition Survey Scale was used to measure participants’ levels of cognition about school bullying (39). The scale comprises 17 items that are designed to assess various forms of bullying cognition, including physical bullying (e.g., “deliberately kicking, pushing or hitting others”); social bullying (e.g., “deliberately excluding others, ostracizing or ignoring others”); verbal bullying (e.g., “scolding or making fun of others”); and other forms of bullying (e.g., “swearing, mocking or threatening others online”). Each item is rated on a 4-point Likert scale, ranging from ‘true’ to ‘completely false,’ where a response of “true” indicates a support of bullying behavior, and a response to one of the other options indicates varying degrees of opposition to or less understanding of bullying behavior. A higher total score reflects a more comprehensive cognition level of school bullying behaviors. In this survey, the internal consistency of the scale, as assessed by Cronbach’s alpha, was determined to be 0.83.

#### 2.2.2 Psychological resilience

The Psychological Resilience Scale was used to measure the level of resilience of primary school students (40). The scale consists of 14 items (e.g., “I am generous with my friends,” “I quickly get over and recover from fear,” “I enjoy dealing with new and unusual situations.”), and the answer to each item is scored on a scale from 1 to 4 points from “completely incongruent” to “completely coincident.” The higher the score is, the greater the level of psychological resilience. In this survey, the Cronbach’s alpha was 0.76.

#### 2.2.3 School bullying

We used the Bullying Behavior Questionnaire to determine the occurrence of school bullying (41). The questionnaire consists of 14 items (Cronbach’s alpha = 0.77), including two scales for victims (e.g., “someone deliberately kicked, pushed or hit me,” “someone given me an unpleasant nickname due to my accent or dress.”), and perpetrators of bullying (e.g., “I intentionally kick, push or hit other students,” “I gave others an unpleasant nickname due to his or her accent or dress.”). Each item is scored on a scale of 0–3 points, where 0 = “not happened,” 1 = “1–2 times,” 2 = “3–4 times,” and 3 = “5 times and more,” for a total score of 42 points (21 points for bullying others and 21 points for being bullied by others).

### 2.3 Data analysis

EpiData 3.0 was used for data entry, and SPSS 25.0 was used for statistical analysis. Age, school bullying cognition, psychological resilience, school bullying perpetration score, and school bullying victimization score were continuous variables. Sex was dichotomized (0 = “male,” 1 = “female”). Grade was a nominal variable (1 = “3rd grade,” 2 = “4th grade,” 3 = “5th grade”). Normally distributed data were described as Mean ± Standard Deviation for statistical description, and the t test was used for comparisons between sexes. The correlations between bullying cognition and psychological resilience and between the bullying perpetration score and the bullying victimization score were analyzed via Pearson correlation analyses. The mediation and moderation models were analyzed by using the PROCESS 3.3 macro program compiled by Hayes after controlling for grade. A total of 5,000 bootstrapping samples were used to calculate the bias-corrected 95% confidence interval (*CI*). First, we tested the association between bullying cognition and school bullying (including school bullying perpetration and school bullying victimization) by assessing psychological resilience using Model 4 of the PROCESS. If the 95% *CI* of a*b did not contain 0, the indirect effect was considered significant. Model 59 of PROCESS was subsequently used to examine the moderated mediation effect of sex, namely, whether sex moderates the direct and indirect effects of bullying cognition and school bullying, including both perpetration and victimization. A 95% CI of the interaction that did not contain 0 indicated that the moderated mediation effect was established (42). Simple slope analysis was used to further analyze the moderating effect of sex. The test level was *α* = 0.05.

## 3 Results

### 3.1 Sociodemographic characteristics of the participants

The average age of the participants was 9.53 ± 0.96 years, and the proportions of males and females were 52.4 and 47.6%, respectively. The percentages of students in the third, fourth and fifth grades were 30.1, 35.0 and 34.9%, respectively.

### 3.2 Mean, standard deviation and correlation analysis of variables

The scores for school bullying victimization, school bullying perpetration, school bullying perception and psychological resilience were 10.31 ± 4.02, 7.92 ± 2.30, 63.57 ± 6.22 and 40.01 ± 8.06, respectively. School bullying victimization was significantly negatively correlated with psychological resilience and bullying cognition. There were also significant negative correlations between school bullying perpetration and psychological resilience and between school bullying perpetration and cognition. There was a significant positive correlation between psychological resilience and bullying cognition (Table 1).

**Table 1:** Mean, standard deviation and correlation analysis of variables.

Variables	^−^x ± s	Min	Max	1	2	3	4
1. Bullying victimization	10.31 ± 4.02	7	33	1			
2. Bullying perpetration	7.91 ± 2.30	7	31	0.362^***^	1		
3. Resilience	40.01 ± 8.06	14	56	−0.148^***^	−0.136^***^	1	
4. Bullying cognition	63.57 ± 6.22	17	110	−0.099^***^	−0.157^***^	0.213^***^	1

^***^p < 0.001.

### 3.3 Comparison of school bullying, bullying cognition and psychological resilience scores among primary school students by sex

There were statistically significant differences in school bullying victimization, school bullying perpetration, school bullying cognition and psychological resilience scores among primary school students of different sexes. Compared with girls, boys had higher scores for school bullying victimization and school bullying perpetration and lower scores for school bullying cognition and psychological resilience (Supplementary Table S1).

### 3.4 Bullying cognition and school bullying: the mediating effect of psychological resilience and the moderating effect of sex

**Figure 2:**
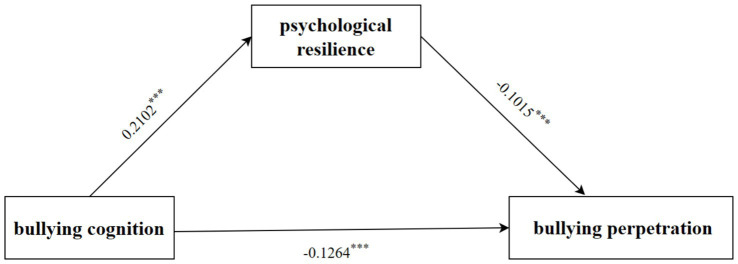
Roadmap of the mediating effect of psychological resilience on the relationship between bullying cognition and bullying perpetration. ***p < 0.001.

As shown in Figure 2, bullying cognition significantly predicted school bullying perpetration (*β* = −0.1478, *p* < 0.001), and the direct predictive effect of bullying cognition on school bullying perpetration was still significant after adding psychological resilience as the mediating variable (*β* = −0.1264, *p* < 0.001). The positive predictive effect of bullying cognition on resilience was significant (*β* = 0.2102, *p* < 0.001), and the negative predictive effect of resilience on school bullying perpetration was also significant (*β* = −0.1015, *p* < 0.001). Psychological resilience partially mediated the relationship between school bullying cognition and school bullying perpetration (*β* = −0.0079, 95% *CI*: −0.0104 to −0.0055). More details are shown in Supplementary Table S2.

**Figure 3:**
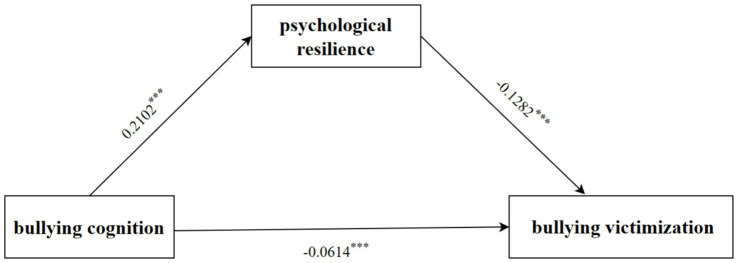
Roadmap of the mediating effect of psychological resilience on the relationship between bullying cognition and bullying victimization. ***p < 0.001.

The mediating effect of psychological resilience on the relationship between bullying cognition and school bullying victimization is shown in Figure 3. Bullying cognition significantly predicted school bullying victimization (*β* = −0.0884, *p* < 0.001), and the direct predictive effect of bullying cognition on school bullying victimization was still significant after adding psychological resilience as the mediating variable (*β* = −0.0614, *p* < 0.001). The positive predictive effect of bullying cognition on resilience was significant (*β* = 0.2102, *p* < 0.001), and the negative predictive effect of resilience on school bullying victimization was also significant (*β* = −0.1282, *p* < 0.001). Psychological resilience mediated the indirect effect of school bullying cognition on school bullying victimization through psychological resilience (mediator) (*β* = −0.0174, 95% *CI*: −0.0219 to −0.0132). More details are shown in Supplementary Table S3.

The moderating effect of sex is shown in Table 2. The interaction effect between bullying cognition and sex was statistically significant for school bullying perpetration (*β* = 0.0383, *p* < 0.001) and victimization (*β* = 0.0040, *p* < 0.001). The results of simple slope analysis showed that with the improvement of bullying cognition, both girls and boys experienced a decrease in school bullying perpetration (girls, *β* = −0.03, *p* < 0.001; boys, *β* = −0.06, *p* < 0.001) and victimization (girls, *β* = −0.02, *p* < 0.001; boys, *β* = −0.06, *p* = 0.017), but the decline in school bullying was greater for boys than for girls (Supplementary Figures S1, S2). The interaction effect of psychological resilience and sex on school bullying perpetration and victimization was not significant.

**Table 2:** Test of the moderation of the relationship between the bullying cognition and school bullying.

Predictive variable	Model 1^a^		Model 2^b^		Model 3^c^	
	*β*	*t*	*β*	*t*	*β*	*t*
Bullying cognition	0.2710	16.3475^***^	−0.0384	−4.528^***^	−0.0451	−9.3483^***^
Sex	0.8510	4.1401^***^	−0.8829	−8.5718^***^	−0.5002	−8.5331^***^
Grade	−0.2198	−1.7233	−0.1799	−2.8186^**^	−0.0338	−0.9295
Resilience			−0.0639	−9.7974^***^	−0.0285	−7.6951^***^
Bullying cognition * Sex	−0.0339	−1.0205	0.0400	2.3538^*^	0.0383	3.9637^***^
Resilience * Sex			−0.0118	−0.9045	0.0139	1.8749
*R*	0.2204		0.2022		0.2256	
*R* ^2^	0.0486		0.0409		0.0509	
*F*	75.3017^***^		41.9028^***^		52.6967^***^	

Model 1^a^: dependent variable is resilience; Model 2^b^: dependent variable is victimization; Model 3^c^: dependent variable is perpetration. *p < 0.05, **p < 0.01, ***p < 0.001.

## 4 Discussion

This study showed that school bullying cognition can reduce the prevalence and risk of school bullying. Psychological resilience partially mediated the relationship between school bullying cognition and school bullying perpetration and victimization; similarly, the greater the respondent’s resilience was, the less involvement he or she was in bullying behavior. Sex moderated the relationship between school bullying cognition and school bullying perpetration behavior. With the same level of school bullying cognition, boys were more likely to engage in school bullying perpetration than girls were. Our findings provide new references and perspectives for revealing the causes of school bullying and preventing and reducing this behavior.

This study found that the average resilience scores of participants were lower than those reported by Liu (43). There is a strong link between students’ resilience and a variety of environmental factors, especially the home environment, parenting styles, and school climate (44). Given that elementary school students are in a critical period of physical and mental growth, creating a warm and supportive family environment, having a caring and responsive teaching staff, and promoting positive peer interactions are essential to enhancing their resilience. Further analysis showed that compared with previous studies in Shenzhen (45), primary school students in this study had higher level of perceptions to school bullying, which may be due to the difference in the timing of the survey sample or may reflect the increased perception of bullying in schools among the current primary school student population. Therefore, bolstering primary students’ resilience and perceptions of bullying necessitates joint efforts and continuous focus from families, schools, and society.

This study indicated that bullying cognition is negatively correlated with school bullying behavior and victimization, and these results are consistent with Hypothesis 1. This finding is in line with the findings of several studies showing that higher levels of school bullying are associated with lower levels of involvement in school bullying behaviors, both in terms of perpetration and victimization (46, 47). We speculate that people with higher levels of school bullying cognition can better understand the adverse effects of school bullying and may have greater problem solving, interpersonal and communication skills. Individuals can think from multiple perspectives when they encounter school bullying and are able to adopt more appropriate ways to address school bullying to reduce their involvement in school bullying, which is consistent with cognitive-behavioral theory (10). In addition, previous studies have shown that improving or correcting inappropriate school bullying cognition among children and adolescents can effectively reduce the occurrence of school bullying, both as perpetrators and victims (14, 15). This finding also showed that reducing the occurrence of school bullying by enhancing students’ correct cognitions of school bullying is effective and feasible. In addition, studies have shown that students’ cognitions of school bullying are influenced by external factors such as school climate, teachers, family, and community (48). Therefore, improving students’ cognitions of school bullying requires the participation of schools, teachers, families, and society as a whole.

Our study showed that psychological resilience partially mediated the relationship between pupils’ school bullying cognitions and school bullying perpetration and victimization, which is consistent with Hypothesis 2. Individuals with high bullying cognition and low psychological resilience are unable to actively mobilize their own resources to reasonably address the bullying situation they are currently facing and are more likely to react negatively, which leads to negative outcomes; these individuals become the perpetrator or victim of school bullying, manifesting as the separation of cognition and practice in school bullying. In contrast, individuals with high bullying cognition and high psychological resilience are more likely to respond positively, such as by handling the situation calmly and seeking help, which manifests as alignment between cognition and behavior in school bullying. Bullying cognition was positively correlated with psychological resilience, and previous studies also support this conclusion (16, 25). Our results also show that students with higher levels of psychological resilience are less likely to be involved in school bullying, as has been demonstrated among students in many countries (21, 49). These studies conclude that enhancing psychological resilience in adolescents effectively reduces students’ involvement in school bullying, both as perpetrators and victims. Factors such as a good family atmosphere, social support and other factors can also improve the level of psychological resilience and reduce adverse outcomes after bullying (26). In conclusion, the higher student cognition of bullying is, the greater the level of psychological resilience, the greater the ability of students to handle adversity positively, and the less likely they are to participate in school bullying, whether as perpetrators and/or victims. Therefore, to reduce the occurrence of school bullying, on the one hand, primary school students’ understanding of school bullying should be improved. Peer interaction and anti-bullying programs can be implemented to provide students with knowledge about bullying in schools and effective strategies for preventing it, thus reducing the occurrence of school bullying. On the other hand, we can improve the psychological resilience of primary school students. Schools can conduct mental health programs to teach students interpersonal and emotional management skills, promoting positive coping strategies and enhancing their mental resilience. Schools and parents can create a good educational and living environment for their children, give students more care and attention, and create a harmonious and relaxed atmosphere, which also helps to improve students’ psychological resilience.

This study also revealed that sex moderates the relationship between school bullying cognition and school bullying perpetration and victimization behavior. Specifically, the predictive effects of school bullying cognition on bullying perpetration and victimization were more pronounced for boys than for girls. In other words, even with the same level of school bullying cognition, boys were more likely to engage in school bullying than girls were, which is partly consistent with Hypothesis 3. Several explanations have been proposed for this difference. First, these differences may be related to biological differences between boys and girls. Boys may be more involved in violent behavior because of the level of hormones in their bodies (50). Second, this difference may be due to the different requirements for male and female behavior under traditional Chinese culture norms. Generally, parents and teachers educate their children by encouraging boys to be brave, non-retreating, lively, etc., and girls to be gentle, restrained and quiet. Therefore, when faced with stressful conditions such as school bullying, boys are more likely to be involved than girls under the guidance of previous education. Finally, this may also be caused by guidance from the online environment and video games; boys are more attracted to violent online games, movies and TV than girls are, which makes boys more likely to participate in violent conflicts or bullying behavior (50). While the negative predictive effect of bullying cognition on school bullying was stronger for boys, this negative predictive effect was also significant for girls. The above results suggest that although there are some differences between sexes, the positive effects of bullying cognition are remaining prevalent. While bullying cognition can influence the prevalence of school bullying through psychological resilience, the results of this study did not reveal a moderating effect of sex in the mediating model. In other words, there were no sex differences in the relationship between bullying cognition and psychological resilience or between psychological resilience and school bullying. This finding suggests that overcoming stereotypes such as school bullying interventions requires only focusing on boys and that boys and girls should be treated equally to reduce the prevalence of bullying in schools when they proceed with psychological resilience interventions. The results of this study showed that the number of effect indicators was low, which may be related to the study population. Our research subjects were primary school students, whose physiological, psychological, and behavioral development is still in a critical shaping period. Therefore, these findings may be less pronounced than those in secondary school students, college students, or adults. Moreover, the primary school stage is a critical period for shaping psychological and cognitive behavior that parents and schools should pay attention to.

This study has several limitations. First, the prevalence of school bullying was self-reported by the respondents, and there may have been information bias. Second, this study did not assess other protective factors, such as school climate, self-efficacy, self-esteem and teachers’ school bullying cognitions, which have also been shown to influence the occurrence of school bullying. Future research could examine the relationships between psychological resilience, student bullying cognition, and bullying behavior after adjusting for additional control variables. Third, this was a cross-sectional study, and it was difficult to infer causality among variables. However, as one of the few studies focusing on school bullying among Chinese primary school students, the results of this study also provide a reference point for reducing the incidence of school bullying. In the future, the relationships among these three variables can be further explored and validated in longitudinal studies.

## 5 Conclusion

In this study, we explored the possibility that psychological resilience functions as a protective factor against school bullying behaviors. The results showed that psychological resilience mediated the relationship between bullying cognition and school bullying behaviors. The study also revealed that the effect of school bullying cognition on school bullying perpetration was more pronounced among boys. The findings of this study have practical relevance and provide valuable insight into the relationships among school bullying cognition, psychological resilience and school bullying behaviors among primary school students. Cultivating and improving the cognition of school bullying and psychological resilience among primary school students is beneficial for reducing school bullying and thus should be recognized and promoted by education supervision departments, schools, families and the whole society. The effect of school bullying cognition on school bullying perpetration was more pronounced among boys. Schools and families may need to increase their cognition of bullying to decrease its prevalence.

## Data Availability

The raw data supporting the conclusions of this article will be made available by the authors, without undue reservation.
