# Supplementary material for: The impact of bullying cognition on school bullying among Chinese primary school students: a moderated mediation model of resilience and sex
Source: Front Public Health. 2024 Oct 16;12:1470322. doi: 10.3389/fpubh.2024.1470322 (PMC11525983; doi:10.3389/fpubh.2024.1470322)
Supplement: Supplementary file 1 [file Table_1.docx]

Supplementary Material

# Supplementary Tables

| **Table** **S1.** Comparison of school bullying, bullying cognition and psychological resilience score among primary school students of different sexes | | | |
| --- | --- | --- | --- |
| Score of bullying victimization | Score of bullying perpetration | Bullying cognition | Resilience |
| 10.78±4.29 | 8.19±2.66 | 63.14±6.42 | 39.49±8.17 |
| 9.79±3.62 | 7.62±1.77 | 64.05±5.97 | 40.59±7.90 |
| 9.59 | 9.79 | -5.67 | -5.22 |
| < .001 | < .001 | < .001 | < .001 |

| **Table S2.** Test of the mediation of the relationship between the bullying cognition and school bullying perpetration | | | | | | | | |
| --- | --- | --- | --- | --- | --- | --- | --- | --- |
| Variables | Model 1^a^ | |  | Model 2^b^ | |  | Model 3^c^ | |
|  | β | t |  | β | t |  | β | t |
| Bullying cognition | -0.1478 | -11.5115^***^ |  | 0.2102 | 16.4685^***^ |  | -0.1264 | -9.6788^***^ |
| Sex | -0.1131 | -8.8318^***^ |  | 0.0526 | 4.1276^***^ |  | -0.1078 | -8.4455^***^ |
| Grade | -0.0087 | -0.6758 |  | -0.0222 | -1.7451 |  | -0.0109 | -0.8561 |
| Resilience |  |  |  |  |  |  | -0.1015 | -7.7919^***^ |
| R | 0.1932 |  |  | 0.2200 |  |  | 0.2171 |  |
| R^2^ | 0.0373 |  |  | 0.0484 |  |  | 0.0472 |  |
| F | 76.2796^***^ |  |  | 100.0544^***^ |  |  | 72.9673^***^ |  |
| Indirect Effect | ab=-0.0079 | |  | Bootstrap SE=0.0012 | |  | Bootstrap 95% CI: -0.0104—0.0055 | |
| Note: Model 1^a^: dependent variable is perpetration, independent variable is bullying cognition, and control variables are grade and sex; Model 2^b^: dependent variable is resilience, independent variable is bullying cognition, and control variables are grade and sex; Model 3^c^: dependent variable is perpetration, independent variable is bullying cognition, and control variables are grade, sex and resilience; ****p* < 0.001. | | | | | | | | |

| **Table S3.** Test of the mediation of the relationship between the bullying cognition and school bullying victimization | | | | | | | | |
| --- | --- | --- | --- | --- | --- | --- | --- | --- |
| Variables | Model 1^a^ | |  | Model 2^b^ | |  | Model 3^c^ | |
|  | β | t |  | β | t |  | β | t |
| Bullying Cognition | -0.0884 | -6.8398^***^ |  | 0.2102 | 16.4685^***^ |  | -0.0614 | -4.6860^***^ |
| Sex | -0.1162 | -9.0146^***^ |  | 0.0526 | 4.1276^***^ |  | -0.1095 | -8.5477^***^ |
| Grade | -0.0325 | -2.5207^*^ |  | -0.0222 | -1.7451 |  | -0.0353 | -2.7630^**^ |
| Resilience |  |  |  |  |  |  | -0.1282 | -9.8044^***^ |
| R | 0.1559 |  |  | 0.2200 |  |  | 0.1999 |  |
| R^2^ | 0.0243 |  |  | 0.0484 |  |  | 0.0400 |  |
| F | 49.0126^***^ |  |  | 100.0544^***^ |  |  | 61.384^***^ |  |
| Indirect Effect | ab=-0.0174 | |  | Bootstrap SE=0.0022 | |  | Bootstrap 95% CI: -0.0219--0.0132 | |
| Note: Model 1^a^: dependent variable is victimization, independent variable is bullying cognition, and control variables are grade and sex; Model 2^b^: dependent variable is resilience; independent variable is bullying cognition, and control variables are grade and sex; Model 3^c^: dependent variable is victimization, independent variable is bullying cognition, and control variables are grade, sex and resilience; **p* < 0.05; ***p* < 0.01; ****p* < 0.001. | | | | | | | | |
